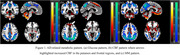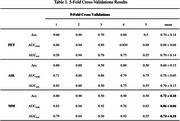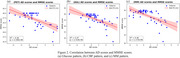# Cerebral Blood Flow and Glucose Metabolic Patterns in Alzheimer's Disease

**DOI:** 10.1002/alz70861_108035

**Published:** 2025-12-23

**Authors:** Jiayi Zhang, Yong Zhang, Weiying Dai, Li Zhao

**Affiliations:** ^1^ Zhejiang University, Hangzhou, Zhejiang China; ^2^ GE Healthcare, Shanghai, Shanghai China; ^3^ Binghamton University, Binghamton, NY USA

## Abstract

**Background:**

Metabolism alternations of brain may reveal the early functional changes in the Alzheimer's disease (AD). A Scaled Subprofile Model‐based method successfully investigated the Parkinson's disease (PD)‐related metabolic Pattern using ASL‐MRI or FDG‐PET individually. We proposed a multi‐modality (MM) pattern, which demonstrated improved accuracy in PD diagnosis. In this work, an AD‐related multi‐modality pattern is proposed and its performance was evaluated.

**Method:**

18 AD patients (75.16±6.09 years old, 6 female), 38 MCI (72.66±7.14 years old, 16 female), and 32 controls (75.50±6.82 years old, 13 female) were retrospectively selected from the ADNI dataset based on the availability of FDG‐PET, MMSE, and ASL‐MRI from GE scanners.

All images were normalized to MNI space and smoothed using SPM12. Cerebral blood flow (CBF) information was extracted from ASL‐MRI. Three AD‐related patterns were constructed. First, the glucose‐ and CBF‐related patterns were constructed individually by linear combinations of the principal components (PCs) that represented the first 60% variance and showed significant differences (*p* <0.05) between CN and AD. Then, the MM pattern was constructed by combining glucose and CBF PCs into a linear model with weights optimized through logistic regression to minimize the Akaike information criterion.

**Result:**

All AD‐related patterns, Figure 1, showed decreased metabolism (blue) in the precuneus, cingulate cortex, occipital, parietal, temporal lobe, and increased metabolism (red) in cerebellum. The CBF‐related pattern showed increased metabolism in the putamen and frontal regions, which were adapted into the proposed MM pattern.

The most distinguishable scores between CN and AD was found in MM‐related pattern (*p* =2.40e‐05 in MM, *p* =6.84e‐05 in Glucose, and *p* =3.47e‐04 in CBF). Although the patterns were established based on the AD and CN, the scores of MCI fell between CN and AD and showed significant differences from AD (*p* =0.006 in PET, 0.015 in ASL, and 0.003 in MM). The MM‐related pattern provided 5.71% higher AUC and 2.86‐5.89% higher accuracy in the five‐fold cross‐validations, Table 1, and improved correlation with the MMSE scores, Figure 2.

**Conclusion:**

The proposed multi‐modality pattern yielded more distinguishable score, superior accuracy, higher correlations with MMSE scores compared to the patterns established based on ASL‐MRI or FDG‐PET individually.